# Retraction: MicroRNA-20b (miR-20b) Promotes the Proliferation, Migration, Invasion, and Tumorigenicity in Esophageal Cancer Cells via the Regulation of Phosphatase and Tensin Homologue Expression

**DOI:** 10.1371/journal.pone.0262879

**Published:** 2022-01-19

**Authors:** The PLOS ONE Editors

Following the publication of this article [1], concerns were raised about the reported results and methods. Specifically,

- In Fig 6A, there appear to be repetitive elements within the following panels:
  ○ Eca-109 0h miR-20b mimics + PTEN vector
  ○ Eca-109 24h blank control
  ○ KYSE-150 24h miR-20 inhibitor + PTEN siRNA
  ○ KYSE-150 24h miR-20 inhibitor + control siRNA
- In Fig 7A, there appear to be repetitive elements within and/or between the following panels:
  ○ Eca-109 blank control
  ○ Eca-109 miR-20b mimics-NC
  ○ Eca-109 miR-20b mimics
  ○ KYSE-150 blank control
  ○ KYSE-150 miR-20b inhibitor-NC
  ○ KYSE-150 miR-20 inhibitor
- Forward and reverse primers used in RT-qPCR for amplification of miR-20b may not align with the miR-20b transcript in the human mRNA reference dataset.
- The SKGT-5 cell line used in this study is described as a human esophageal carcinoma cell line, however this cell line has been shown to be derived from SK-GT-2, a gastric fundus carcinoma cell line.

The PLOS Publication Ethics team investigated these issues and asked the authors to comment on these concerns. The authors did not respond to our inquiries.

The concerns remain unresolved and call into question the reliability of the reported results. Therefore, the *PLOS ONE* Editors retract this article. We regret that the issues in this article were not identified prior to publication.

The authors either did not respond directly or could not be reached.
